# Stable aneuploid tumors cells are more sensitive to TTK inhibition than chromosomally unstable cell lines

**DOI:** 10.18632/oncotarget.16213

**Published:** 2017-03-15

**Authors:** Marion A.A. Libouban, Jeroen A.D.M. de Roos, Joost C.M. Uitdehaag, Nicole Willemsen-Seegers, Sara Mainardi, Jelle Dylus, Jos de Man, Bastiaan Tops, Jules P.P. Meijerink, Zuzana Storchová, Rogier C. Buijsman, René H. Medema, Guido J.R. Zaman

**Affiliations:** ^1^ Netherlands Translational Research Center B.V., Oss, The Netherlands; ^2^ Netherlands Cancer Institute, Amsterdam, The Netherlands; ^3^ Department of Pathology, Radboud University Medical Center, Nijmegen, The Netherlands; ^4^ Princess Máxima Center for Pediatric Oncology, Utrecht, The Netherlands; ^5^ University of Kaiserslautern, Kaiserslautern, Germany

**Keywords:** TTK, Mps1, kinase inhibitor, chromosome instability, aneuploidy

## Abstract

Inhibition of the spindle assembly checkpoint kinase TTK causes chromosome mis-segregation and tumor cell death. However, high levels of TTK correlate with chromosomal instability (CIN), which can lead to aneuploidy. We show that treatment of tumor cells with the selective small molecule TTK inhibitor NTRC 0066-0 overrides the mitotic checkpoint, irrespective of cell line sensitivity. In stable aneuploid cells NTRC 0066-0 induced acute CIN, whereas in cells with high levels of pre-existing CIN there was only a small additional fraction of cells mis-segregating their chromosomes. In proliferation assays stable aneuploid cells were more sensitive than cell lines with pre-existing CIN. Tetraploids are thought to be an intermediate between diploid and unstable aneuploid cells. TTK inhibitors had the same potency on post-tetraploid and parental diploid cells, which is remarkable because the post-tetraploids are more resistant to mitotic drugs. Finally, we confirm that the reference compound reversine is a TTK inhibitor and like NTRC 0066-0, inhibits the proliferation of patient-derived colorectal cancer organoids. In contrast, treatment with TTK inhibitor did not reduce the viability of non-proliferating T cell acute lymphoblastic leukemia cells samples. Consequently, TTK inhibitor therapy is expected to spare non-dividing cells, and may be used to target stable aneuploid tumors.

## INTRODUCTION

Chromosomal instability (CIN), which manifests as a constant change in karyotype, is a hallmark of tumor malignancy and is thought to be one of the main causes of ‘aneuploidy’, a stable state of an imbalanced chromosome number [1–3]. Aneuploidy and CIN both have been correlated with poor patient outcome in multiple cancer types, including lung, breast and colon cancer [4, 5]. The selective advantage of CIN to cancer growth is thought to derive from its effect on intra-tumor heterogeneity, facilitating the selection of chemotherapy resistant clones [6]. In parallel, in aneuploid cells, an abnormal chromosome count may deregulate cancer pathways or confer therapy resistance by duplication or loss of specific genes [7–9]. Paradoxically, induction of aneuploidy decreases fitness of non-transformed cells [10]. This suggests that cancer cells have acquired mutations that help them to cope with the detrimental effects of aneuploidy or CIN and that these mutations could be targeted at a molecular level. However, the molecular mechanisms that drive or suppress CIN remain elusive in cancer. At least it is clear that there is no single molecular mechanism that can explain CIN in all human cancers. Mutations in the gene for *BUB1*, a component of the spindle assembly checkpoint (SAC) [11], can induce CIN, but these mutations are infrequent in human tumors. *TP53* gene mutations [12] and mutations in components of the Wnt pathway, such as APC [13], can contribute to CIN in cell lines, but alone are insufficient [12, 13]. However, combined loss of *TP53* and *APC* gives rise to extensive CIN in intestinal organoids [14].

Various strategies have been proposed to target aneuploidy or CIN. One approach is to exploit the cellular stress-state [1, 7] and resulting DNA damage [15] caused by chromosome segregation errors. Another approach exploits the high activation of the SAC in many aneuploid and CIN cells. It has been suggested that because of the abnormal chromosome number, such cells are highly dependent on this checkpoint [2, 16]. Inhibition of the SAC will therefore selectively induce chromosome mis-segregation and cause cell death in aneuploid or CIN cell lines [17], or tumors [18]. Among the best-described SAC inhibitors are small molecule inhibitors of the protein kinase TTK (often referred to as Mps1). Several TTK inhibitors have been shown to reduce the growth of xenografts of human cancer cell lines from diverse tumor tissue origin in mice [18–24]. Furthermore, in an immunocompetent mouse model of triple-negative breast cancer (TNBC) [18], and in patient-derived xenograft models [22] TTK inhibitors increased the efficacy of taxane chemotherapy [18, 22]. In this context, it is encouraging that three TTK inhibitors have entered phase 1 clinical trials for combination therapy with paclitaxel in TNBC or as monotherapy (https://clinicaltrials.gov/).

Definition of the patient population that is most likely to respond based on genomic markers has been imperative to the success of targeted therapies. For example, the use of drugs that selectively target the protein product of the BCR-ABL translocation in chronic myeloid leukemia has revolutionized the treatment of this disease, with five-year survival rates of 90% in treated patients [25]. In the case of TTK inhibitor therapy, the development of a personalized medicine strategy is more challenging. Firstly, mutations in TTK are not detected at high frequency in human cancers, and there is no relationship between mutated or activated TTK and malignancy status known. Secondly, whereas TTK is highly expressed in several cancer types, the relationship between expression level and severity of disease is complex and contradictive. For example, high *TTK* expression correlates with poor prognosis in hepatocellular carcinoma [26] and Her2-positive breast cancer [27], while low *TTK* expression correlates with poor patient outcome in TNBC [27]. Because TNBC targeting is related to chromosomal state [28], we investigated the effects of TTK inhibition in cells with abnormal chromosome states. Thereby, we distinguished between aneuploidy and CIN, and took advantage of the selective and sub-nanomolar potent inhibitor of TTK, NTRC 0066-0 [18]. NTRC 0066-0 potently inhibits the proliferation of human cancer cell lines *in vitro* and reduces tumor growth in mouse cancer models without toxicity [18]. For the first time we studied here the effect of a TTK inhibitor on the viability and proliferation of primary human patient-derived tumor cell samples and organoids. Our data suggest that NTRC 0066-0 only kills proliferating cells and preferably targets stable aneuploid cancer cells.

## RESULTS

### Selection of cell lines for CIN analysis

**Figure 1:**
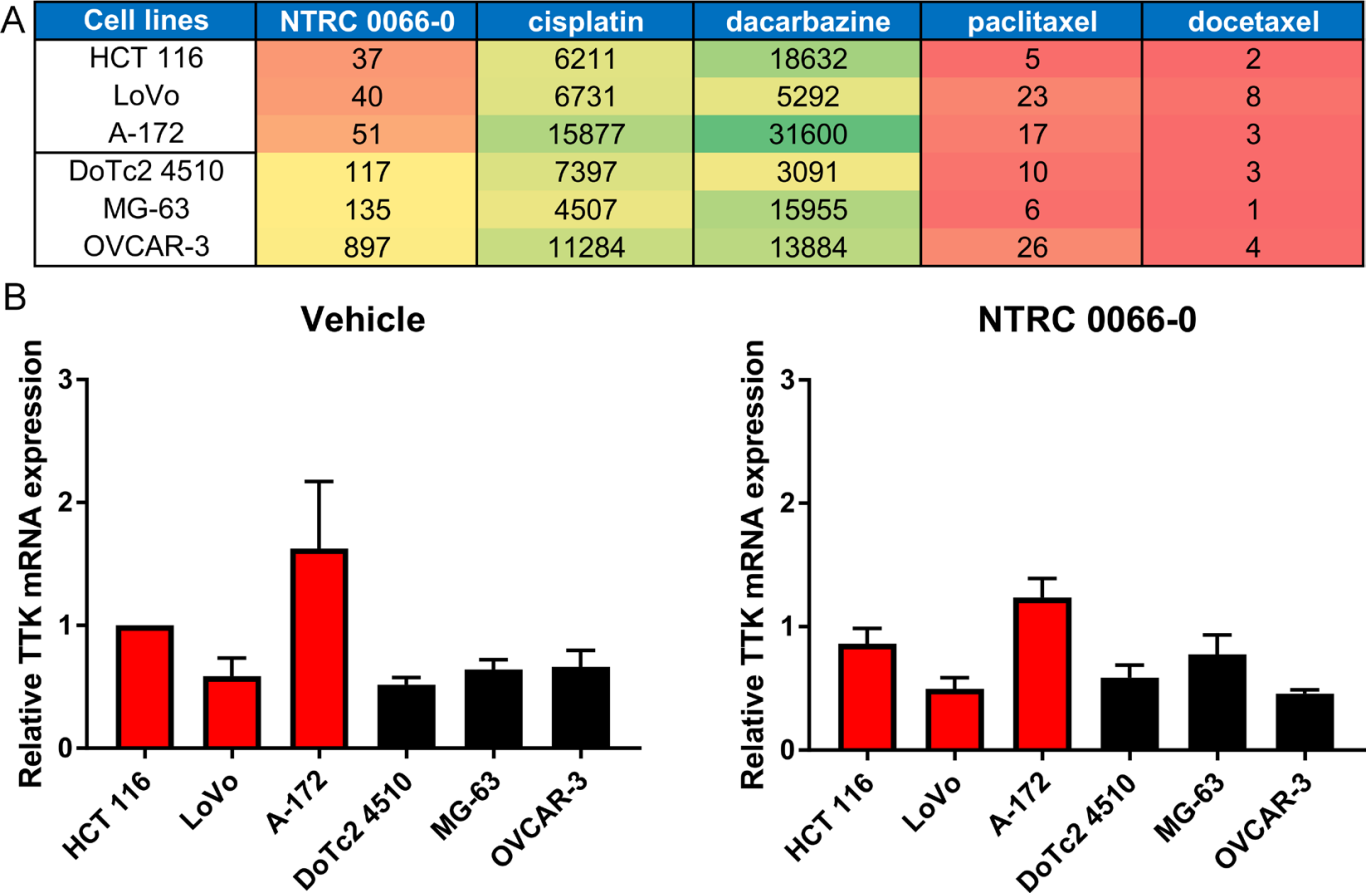
Selection of TTK inhibitor sensitive and resistant cell lines (**A**) Heat map showing the relative sensitivity of six human cancer cell lines to the TTK inhibitor NTRC 0066-0 and four cytotoxic agents. Red relates to sensitivity; green to resistance. The numbers correspond to the IC_50_ values in nM in three day cell proliferation assays [18]. (**B**) TTK mRNA levels relative to the HCT 116 vehicle condition. Cells were treated with vehicle or 100 nM NTRC 0066-0 for 24 hours. Sensitive cell lines are depicted in red and resistant cell lines in black. The average of three independent experiment is shown. Each experiment was performed using three references genes and TTK.

It has been suggested that TTK inhibitor therapy would be in particular effective in cancers characterized by highly unstable genomes [18, 29]. To determine the potential relationship between aneuploidy, CIN and sensitivity to TTK inhibitors, we selected three cell lines that were relatively sensitive to NTRC 0066-0 in a broad cell panel screen [18] and three cell lines that were less sensitive (Figure 1A). The colon carcinoma cell line HCT 116, the colorectal adenocarcinoma cell line LoVo, and the glioblastoma cell line A-172 are relatively sensitive to NTRC 0066-0, having an IC_50_ in three day cell proliferation assays of 37 nM, 40 nM and 51 nM, respectively (Figure 1A). The cervix carcinoma cell line DoTc2 4520, the osteosarcoma cell line MG-63 and the ovary adenocarcinoma cell line OVCAR-3 are less sensitive, having IC_50_s of 117 nM, 135 nM and 872 nM, respectively. For clarity, the two groups of three cell lines are referred to in this study as either ‘sensitive’ or ‘resistant’ (Figure 1A). The same separation of the two groups based on TTK sensitivity was observed in five day proliferation assays and with the structurally different TTK inhibitors MPI-047605, Bay2b and reversine (Supplementary Table 1). Notably, while these cell lines showed different sensitivity to TTK inhibition, their sensitivity to classic chemotherapeutic drugs was similar, such as to the DNA damaging agents cisplatin and dacarbazine, and the microtubule targeting drugs paclitaxel and docetaxel (Figure 1A). To determine whether the sensitivity to NTRC 0066-0 inhibition originates from different levels of TTK, we performed real-time PCR on the six cell lines treated with vehicle or with NTRC 0066-0 (Figure 1B). TTK levels in the six cells lines did not correlate with their sensitivity to TTK inhibition and treatment with NTRC 0066-0 did not modify TTK levels in the six cells lines (Figure 1B). In addition, *in silico* analysis on a sixty-six cell line panel showed no correlation between TTK levels and the potency of NTRC 0066-0 (Pearson correlation of -0.1).

### Chromosomally stable cell lines are more sensitive to TTK inhibition

**Figure 2:**
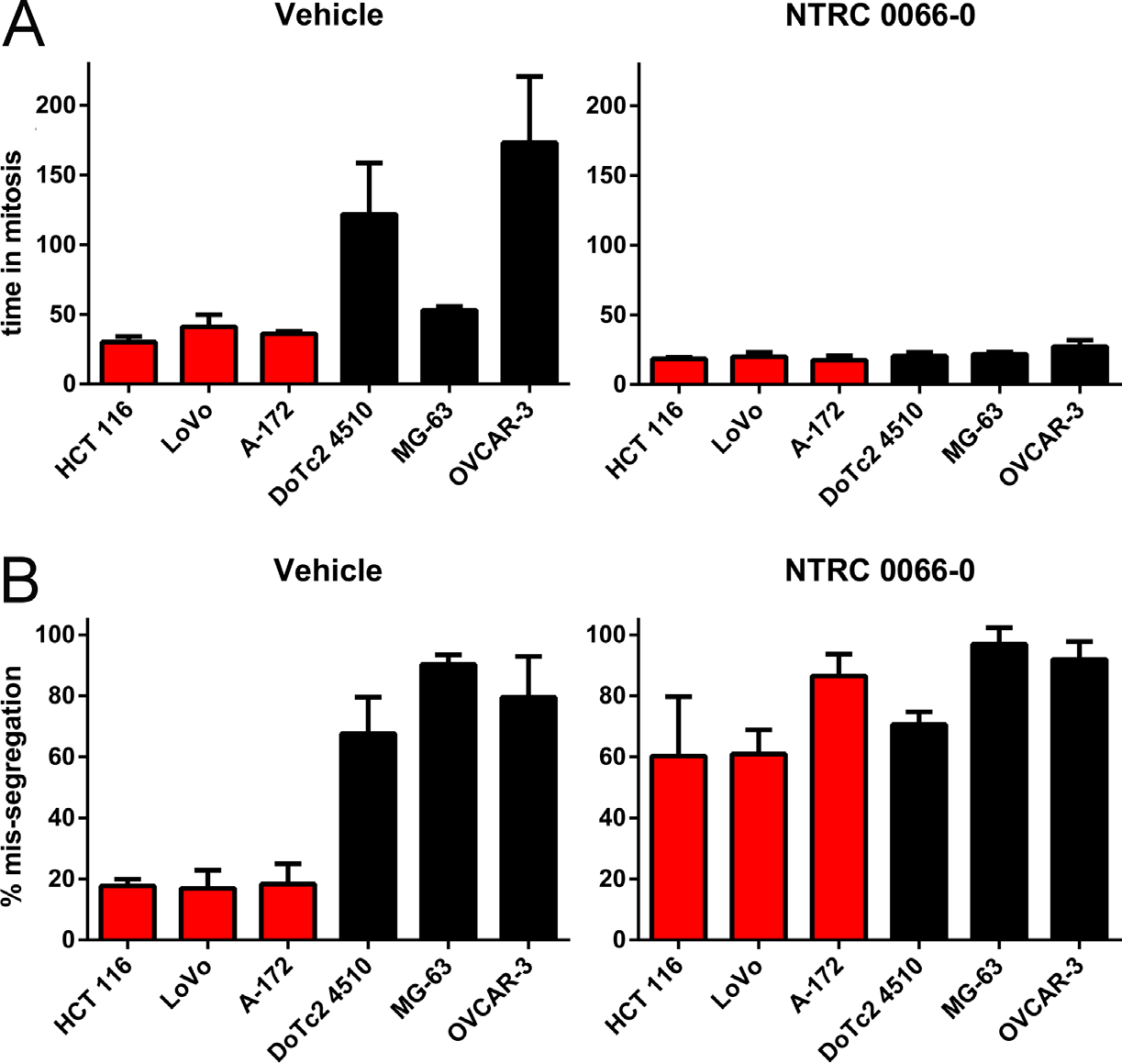
Mitotic timing and mis-segregation in TTK inhibitor sensitive and resistant cell lines Time lapse microscopy analysis of sensitive (red) and resistant (black) cell lines treated with vehicle (left) or with 100 nM NTRC 0066-0 (right). The bar graphs represent means and standard deviation calculated from three independent experiments. For one experiment 36 cells were quantified on average. (**A**) Time in mitosis (from nuclear envelope breakdown to anaphase onset). (**B**) Percentage of cells dividing with mis-segregation.

Whereas TTK inhibition has been shown to induce chromosome mis-segregation and cell death in many different cell types [18–22, 30], it remains unclear why some cells are more sensitive to TTK inhibition than others. To determine whether sensitivity to TTK inhibition is related to the mitotic process and/or chromosome stability, we compared the activity of the mitotic checkpoint and the effect of TTK inhibition on chromosome mis-segregation in cell lines with different sensitivity to NTRC 0066-0 by time-lapse microscopy. Cell lines were treated with 100 nM NTRC 0066-0, or vehicle only (DMSO) (Figure 2). The activity of the mitotic checkpoint was measured by the time between the breakdown of the nuclear envelop and the onset of anaphase (Figure 2A). Under the vehicle-treated control conditions, all sensitive cells were able to accomplish a normal mitosis in approximately 35 minutes (HCT 116, 30 min; LoVo, 41 min; A172, 36 min; Figure 2A and Supplementary Figure 1). In contrast, the three resistant cell lines spent more time in mitosis, *i.e*., almost one hour (MG-63, 53 min), or up to 2 and 3 hours (DoTc2 4510, 122 min; OVCAR-3, 173 min). When treated with NTRC 0066-0, time-in-mitosis reduced to approximately 20 min for all cell lines (Figure 2A and Supplementary Figure 1).

Next, we scored the percentage of cells with mis-segregation during anaphase as an indicator of CIN. In the sensitive cell lines less than 20% of anaphases were defective, whereas in the resistant cell lines at least 60% of anaphases were defective (Figure 2B, Supplementary Figures 1 and 2). Upon treatment with NTRC 0066-0, the number of defective anaphases increased by three-fold or more in the sensitive cell lines (to 60% or more), whereas the number did not notably change in the resistant cell lines (63–71%, Figure 2B, Supplementary Figures 1 and 2). To confirm that inhibition of the kinase domain of TTK acts on the mitotic checkpoint, we compared NTRC 0066-0 treatment with knockdown of the checkpoint protein Mad2. We were able to knockdown Mad2 in all the cell lines except in DoTc2 4510 (Supplementary Figure 3A). Similarly to NTRC 0066-0 treatment, Mad2 knockdown reduced time-in-mitosis in all cell lines (Supplementary Figure 3B) and increased the number of defective mitosis in the sensitive cell lines by three-fold (Supplementary Figure 3C and 3D). Thus, NTRC 0066-0 treatment phenocopies the inactivation of the mitotic checkpoint induced by decreased Mad2 expression.

These data indicate that the difference in TTK inhibitor sensitivity of the cell lines is not due to differences in mitotic checkpoint override. Furthermore, TTK inhibitor IC_50_s anti-correlate with CIN status.

**Figure 3:**
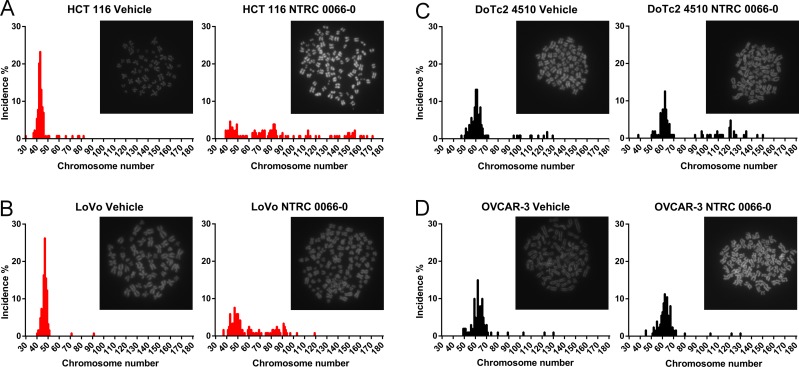
Karyotype analysis of sensitive and resistant cell lines treated with NTRC 0066-0 Karyotype analysis of two sensitive (**A** and **B**) in red) and two resistant (**C** and **D**, in black) cell lines. Cells were synchronized with thymidine block, released, and allowed to progress though mitosis in the presence of vehicle (left) or with 100nM NTRC 0066-0 (right). After 20 hours, the cells were washed and captured in their second mitosis for karyotyping. Karyotype graphs are illustrated by a representative picture depicting DAPI-stained chromosomes. The chromosome counts and their incidence (%) were calculated from the pool of three independent experiments. On average 116 cells were quantified per condition. (A) HCT116. (B) LoVo. (C) DOTC2 4510. D) OVCAR-3.

To characterize the effect of TTK inhibition on the chromosome content of the progeny, mitotic cells were collected after treatment with NTRC 0066-0 or vehicle for 20 hours (See Supplementary Figure 4). The karyotypes of two sensitive and two resistant cell lines were examined (due to technical reasons not all cell lines were processed). The sensitive cell lines LoVo and HCT 116 showed a near-diploid karyotype in the control condition (Figure 3). In contrast, for DoTc2 4510 and OVCAR-3, the modal chromosome number was around 60, and the spread in chromosome number was higher (Figure 3). This indicates that the two cell lines are hypo-triploid and have an unstable karyotype. These results are consistent with the time-lapse analysis (Figure 2) and the information on these cell lines available on the website of the provider of the cell lines, *i.e*., the American Type Culture Collection (ATCC) (www.atcc.org). After treatment with NTRC 0066-0, the sensitive cell lines displayed a much more divergent karyotype than the resistant lines (Figure 3). The chromosome count in the resistant, near-triploid lines was less affected. We only observed a slight increase in chromosome number, indicating that the cells did not lose redundant chromosomes as a coping mechanism at this stage. Taken together, we conclude that TTK inhibition is effective in aneuploid cells that have no CIN phenotype. Furthermore, treatment with NTRC 0066-0 immediately caused the induction of CIN.

### Drug sensitivity analysis of post-tetraploid cells

**Figure 4:**
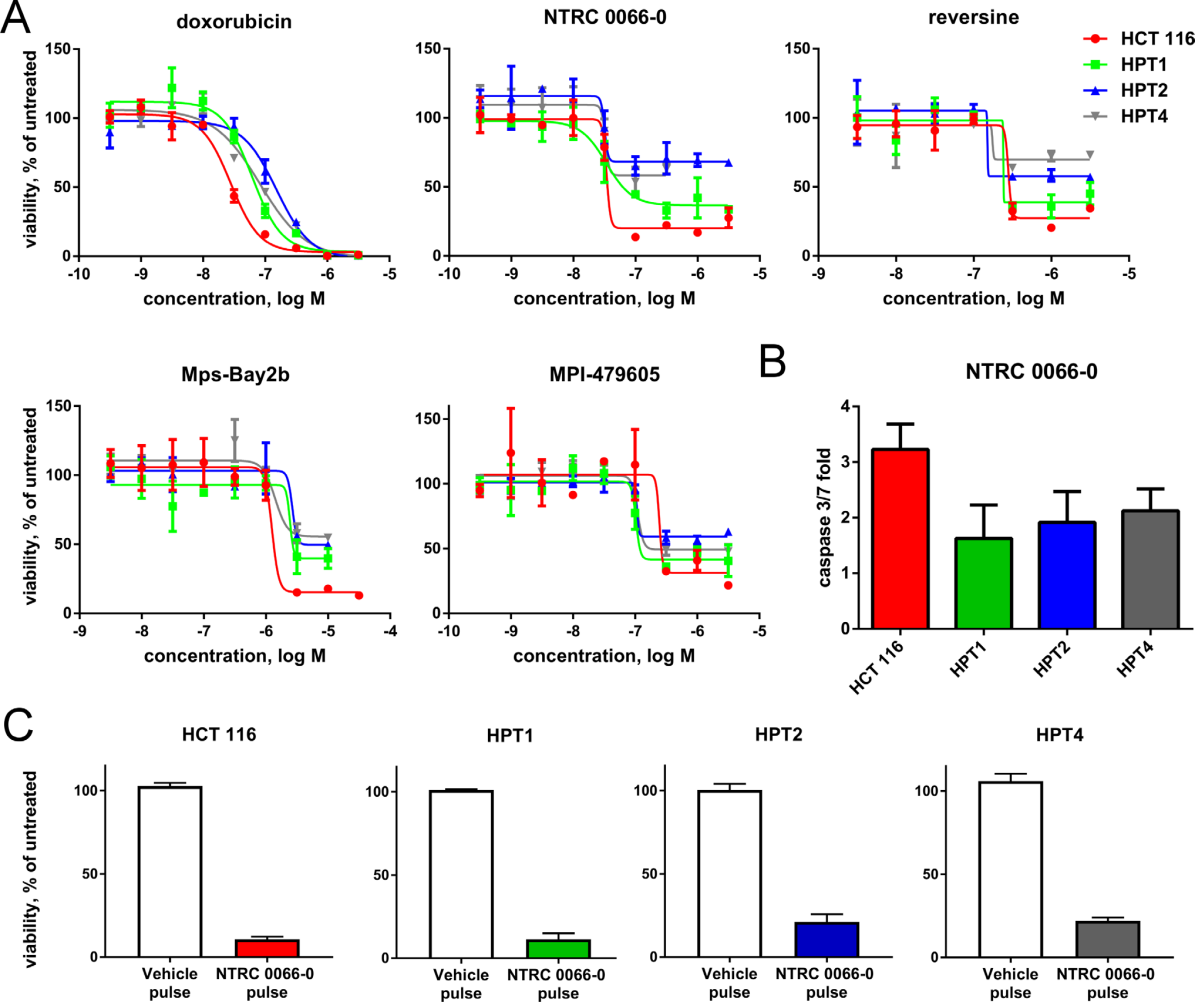
Anti-proliferative activity of TTK inhibitors on HCT 116 diploid and post-tetraploid cells (**A**) Dose-response curves of doxorubicin (control), and the TTK inhibitors NTRC 0066-0, reversine, Mps-Bay2b and MPI-479605 in proliferation assays with near-diploid HCT 116 and post-tetraploid HPT1, HPT2 and HPT4 cells. Cells were treated with compounds for five days. Curves were fitted using the values of three independent experiments. (**B**) Caspase 3/7 activity in cells treated for three days with NTRC 0066-0 (316 nM). Caspase 3/7 activity was calculated as fold increase in comparison to vehicle-treated cells. The bar graphs show the mean and standard deviation from three independent experiments. (**C**) Colony formation assay of cells pre-treated for four days with NTRC 0066-0 (100 nM) or vehicle. Growth was normalized to untreated cells. Quantification of results from three independent experiments.

The resistant cell lines DoTc2 4510, MG-63 and OVCAR-3, are hypo-triploid unstable cell lines (Figures 2, 3; www.atcc.org). Interestingly, such karyotypes are believed to arise from tetraploid cells that inadvertently lost chromosomes [31–33]. Therefore, we wanted to know whether post-tetraploid precursors are also resistant to TTK inhibitors relative to diploid cells. For this analysis, we used post-tetraploid clones derived from the near-diploid, colon carcinoma cell line HCT 116. We have previously shown that the post-tetraploid clones (referred here as ‘tetraploids’) show low level resistance against multiple cytotoxic agents and a number of targeted drugs [34]. In contrast, NTRC 0066-0 inhibited the proliferation of the three post-tetraploids with a potency (IC_50_) similar to that of the parental HCT 116 cell line (Figure 4A; Supplementary Table 2). The same effect was seen with three other TTK inhibitors, *i.e*., MPI-0479605, Mps-Bay2b and reversine. However, the maximum inhibitory effect (efficacy) of the TTK inhibitors was significantly decreased, indicating that the compounds had a cytostatic effect on the post-tetraploid cells (Figure 4A; Supplementary Table 2). It is important to emphasize that the proliferation rate of the post-tetraploids are the same as that of the parental HCT 116 cell line [34].

Since treatment with TTK inhibitor is expected to result in apoptosis, we determined the post-tetraploid cells' ability to undergo apoptosis by measuring caspase 3/7 activation. Treatment with NTRC 0066-0 induced caspase 3/7 activity in both the parental cell line and in HPT1, HPT2 and HPT4. However, the maximum caspase 3/7 activity was significantly higher in the parental line in comparison to the post-tetraploids (Figure 4B). Because the tumour suppressor p53 induces cell cycle arrest following defective mitosis in post-tetraploid cells [35], we examined whether treatment with NTRC 0066-0 drove cells into irreversible arrest instead of apoptosis. Cells were pre-treated for 4 days with NTRC 0066-0, and after compound wash-out, reseeded in a tissue culture plate. Whereas vehicle-treated cells grew, TTK inhibitor pre-treated cells were unable to fully resume proliferation (Figure 4C). Thus, although a fraction of the post-tetraploid cells appears resistant to NTRC 0066-0 in proliferation assays, a large subset has been permanently compromised by NTRC 0066-0 exposure.

**Figure 5:**
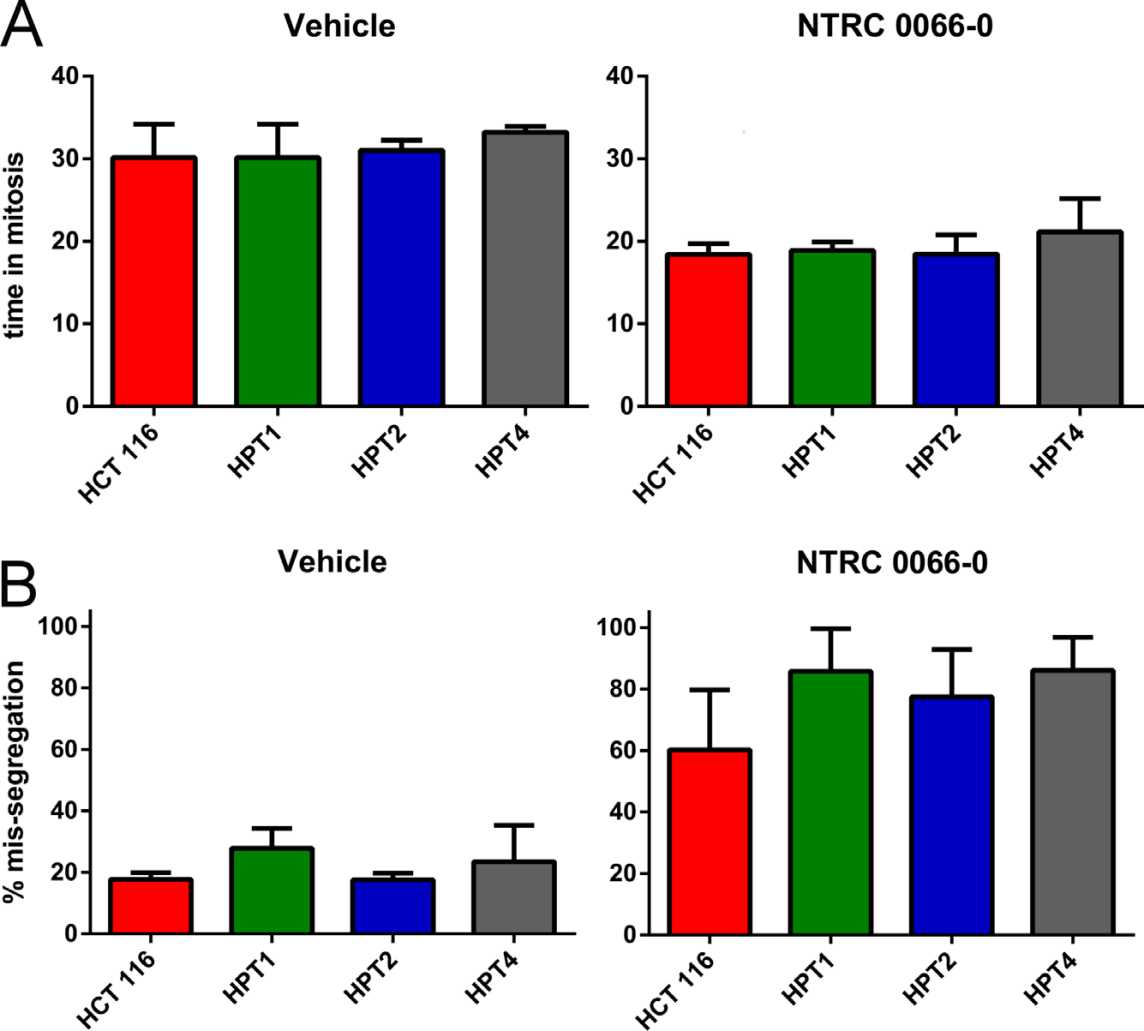
Mitotic timing and mis-segregation in HCT 116 diploid and post-tetraploid cell lines Time lapse microscopy analysis of the post-tetraploid cell lines HPT1 (green), HPT2 (blue) and HPT4 (grey), after treatment with vehicle (left) or 100 nM NTRC 0066-0 (right). The bar graphs show the mean and standard deviation from three independent experiments. For one experiment 36 cells were quantified on average. For clarity, the data of the parental HCT 116 cell line are the same as shown in Figure 2. (**A**) Time in mitosis (from nuclear envelope breakdown to anaphase onset). (**B**) Percentage of cells dividing with mis-segregation.

**Figure 6:**
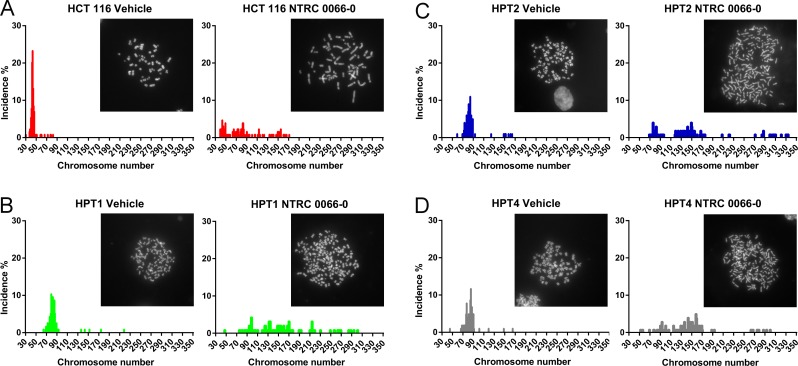
Karyotype analysis of HCT 116 diploid and post-tetraploid cells Karyotype analysis of the post-tetraploid cells HPT1 (**B**, green), HPT2 (**C**, blue) and HPT4 (**D**, grey). The reference karyotype of the parental HCT 116 (**A**) is depicted for a second time (*cf*. Figure 3A) with a broader graph scale. Cells were synchronized with thymidine block, released, and allowed to progress through mitosis in the presence of vehicle (left) or with 100 nM NTRC 0066-0 (right). After 20 hours the cells were washed and captured in their second mitosis for karyotyping. Karyotype graphs are illustrated by a representative picture. The chromosome count and its incidence (%) were calculated from the pool of three independent experiments. On average 97 cells were quantified per condition.

The amount of CIN in HPT1, HPT2 and HPT4, and the effect of NTRC 0066-0 on mitosis were examined by time-lapse microscopy. Whereas tetraploidization had no effect on mitotic timing of vehicle-treated cells (Figure 5A), treatment with NTRC 0066-0 decreased time-in-mitosis to 20 min in the post-tetraploid clones as well as in the parental cell line (Figure 5A). In the post-tetraploids 20% of anaphases were defective (Figure 5B and Supplementary Figure 5), indicating low levels of CIN [34] in comparison to the resistant cell lines. Like in the parental cells, this number was increased three times upon treatment with NTRC 0066-0 (Figure 5B, Supplementary Figure 5). Furthermore, the time-lapse analysis showed that tetraploidization had no effect on the mitotic checkpoint, or the ability of NTRC 0066-0 to induce chromosome mis-segregation. To determine whether an intrinsic property of the post-tetraploid clones was responsible for the reduced efficacy in the cell proliferation assays (Figure 4A), we analysed their karyotypes after exposure to NTRC 0066-0. As previously described [34], non-exposed post-tetraploids are near-tetraploid (Figure 6). Following treatment with NTRC 0066-0, only chromosome gains were noted, no chromosome losses at this stage (Figure 6). This confirms the above observation that TTK inhibitors work in chromosomally stable cell lines, including cells with low levels of CIN like post-tetraploids, through the acute induction of CIN.

**Figure 7:**
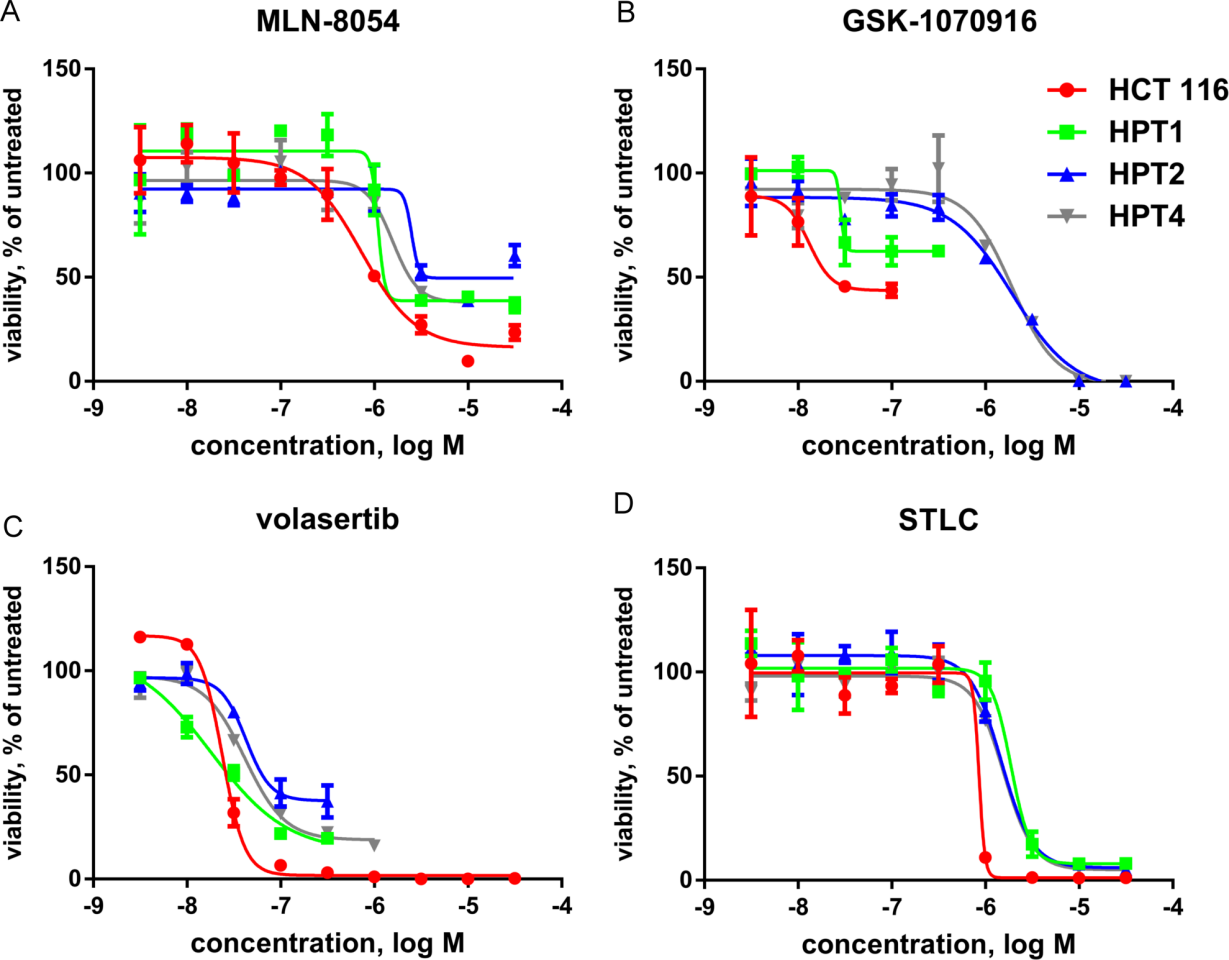
Post-tetraploids are resistant to inhibitors of mitosis Dose-response curves of compounds interfering with mitosis in five day proliferation assays with near-diploid HCT 116 and post-tetraploid HPT1, HPT2 and HPT4 cell lines. Curves were fitted using the values of three independent experiments. (**A**) MLN 8054, an inhibitor of Aurora A. (**B**) GSK1070916, an inhibitor of Aurora B/C. (**C**) volasertib, an inhibitor of Polo-like kinase 1. (**D**) STLC (S-trityl-L-cysteine), an inhibitor of kinesin-5.

We extended our drug sensitivity analysis on the post-tetraploids with other targeted agents interfering with mitosis. We compared the sensitivity of the parental HCT 116 cell line and the post-tetraploids for inhibitors of Aurora A (MLN-8054), Aurora B/C (GSK1070916), Polo-like kinase 1 (volasertib), or kinesin-5 (S-trityl-L-cysteine, STLC). In contrast to TTK inhibitors (Figure 4A), these compounds are less potent on post-tetraploids in comparison to near-diploid HCT 116 cells (Figure 7). However, only for the Aurora kinase inhibitors and STLC the differences in potency (ΔpIC_50_) are statistically significant (Supplementary Table 2). In conclusion, our data suggest that TTK targeting is more selective in inhibiting post-tetraploid cell proliferation than other drugs interfering with mitosis.

### Reversine acts as a selective TTK inhibitor in cells

**Figure 8:**
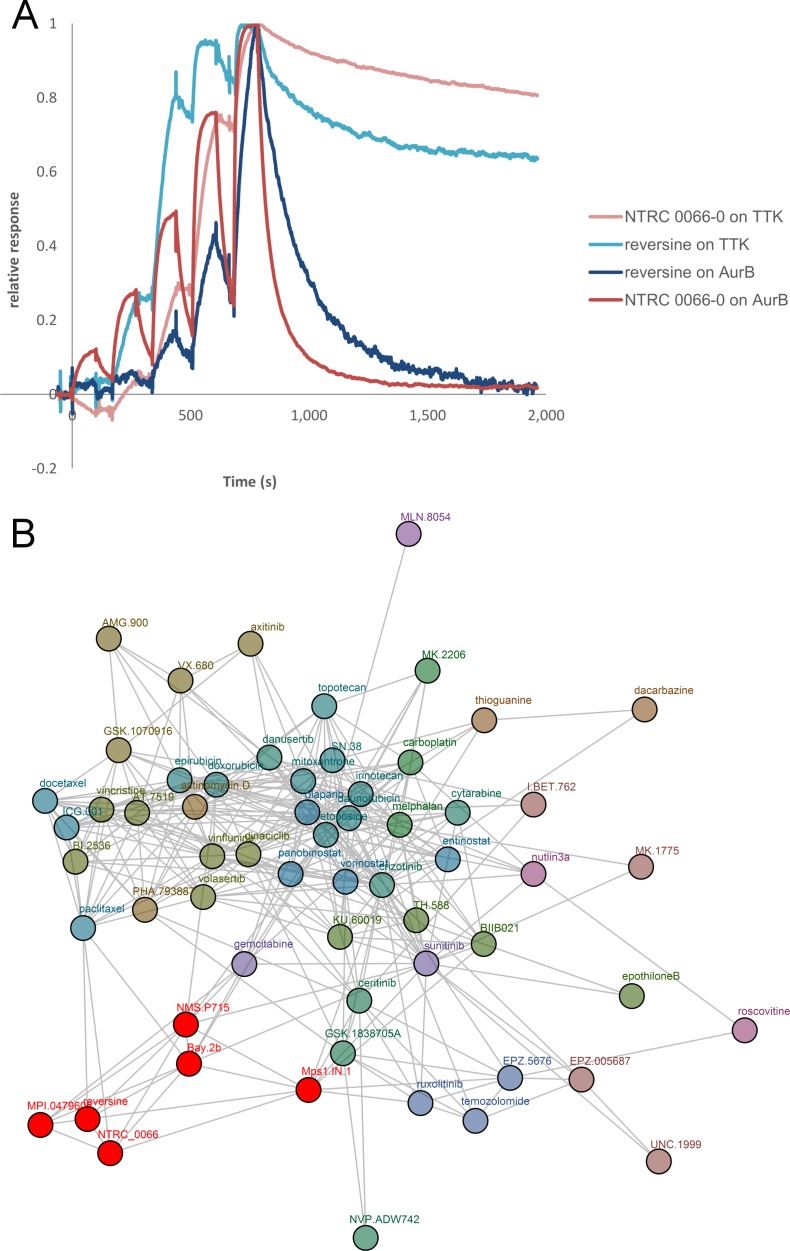
Reversine is a selective TTK inhibitor (**A**) Analysis of the binding of NTRC 0066-0 and reversine to TTK and Aurora B by surface plasmon resonance. An overlay of four sensorgrams is shown of single cycle kinetic experiments with NTRC 0066-0 (red) or reversine (blue) and TTK or Aurora B (AurB) kinase. (**B**) Network tree showing similarity of the profile of reversine with NTRC 0066-0 and other TTK inhibitors in cancer cell line proliferation assays. Connections mean that the profiles of the compounds in 44 or 66 cell line proliferation assays show significant similarity (*i.e*., Pearson correlation ≥ 0.5). A comparative analysis was performed with the profiles of 122 anti-cancer agents [41]. For clarity only compounds within two connections of the investigated compounds (NTRC 0066-0 and reversine) are shown. The TTK cluster containing NTRC 0066-0 and reversine is depicted in red.

When our work was still in progress, Jemáa *et al*. [36] reported that tetraploid HCT 116 cell clones that have been generated independently from the tetraploids used in our study [34, 37], are relatively more sensitive to reversine than parental near-diploid cells. Reversine is a small molecule inhibitor of TTK but also has other activities [37, 38]. Originally, the compound was identified in a phenotypic screen for molecules that can induce stem-cell like phenotypes [38]. Later this property was attributed to its ability to inhibit the mitotic kinase Aurora B [39]. Santaguida *et al*. [40] demonstrated that reversine is also a potent inhibitor of TTK and reported a thirty-five times better potency in kinase assays with TTK in comparison to Aurora B. Both assays were, however, performed at 50 μM ATP whereas the affinity of ATP (K_M,ATP_) for both enzymes differs 100 times and is 160 nM for TTK and 16 μM for Aurora B (Carna Kinase profiling book; www.carnabio.com). Therefore, because of the higher ATP competition in the TTK assay, the selectivity of reversine for TTK over Aurora B may have been underestimated. To be able to relate our data with tetraploids to those of Jemáa *et al*. [36], we determined the precise selectivity of reversine for TTK over Aurora B in surface plasmon resonance (SPR) binding experiments. Figure 8A shows an overlay of SPR sensorgrams of the binding of reversine and NTRC 0066-0 to the kinase domain of TTK and full-length Aurora B. The equilibrium affinity constant (*K*_***D***_) of the interactions were calculated from the association rate (*k*_a_) and dissociation rate (*k*_d_) (*K*_D_ = *k*_d_/*k*_a_). Both reversine and NTRC 0066-0 bind with sub-nanomolar affinity (*K_D_*) to TTK (Table 1). Affinity of reversine for Aurora B is 48 nM; affinity of NTRC 0066-0 for Aurora B is 907 nM (Table 1). Both show an approximately 1000 times selectivity for TTK over Aurora B. NTRC 0066-0 is two times more potent in cell proliferation assays than reversine (Supplementary Table 1), making it the preferred reference TTK inhibitor for cellular studies.

**Table 1:** Kinetic parameters of binding of NTRC 0066-0, reversine, MPI-047605 and Bay 2b to TTK and Aurora B

Enzyme	Inhibitor	*k_a_* (1/Ms)	*k_d_* (1/s)	*K_D_* (M)	*n*
TTK	NTRC 0066-0	3.35E+05	2.81E-04	8.39E-10	8
TTK	reversine	1.60E+06	8.86E-05	5.55E-11	4
TTK	MPI-047605	1.64E+06	9.65E-04	5.90E-10	2
TTK	Bay2b	2.17E+06	2.22E-02	1.03E-08	2
Aurora B	NTRC 0066-0	1.34E+04	1.22E-02	9.07E-07	2
Aurora B	reversine	1.00E+05	4.84E-03	4.81E-08	2
Aurora B	MPI-047605	9.55E+03	2.91E-01	3.05E-05	2
Aurora B	Bay2b	3.57E+04	2.08E-02	5.85E-07	2

Values are geometric averages of 2 to 8 independent measurements (*n*).

To determine the most likely mechanism of how reversine acts on cells, we compared its profile in cell proliferation assays on a panel of sixty-six cancer cell lines (Oncolines^™^) with those of 122 clinical and pre-clinical anti-cancer agents, including several selective TTK and Aurora kinase inhibitors [41]. The results of this comparative analysis are displayed in the network tree in Figure 8B. TTK inhibitors and reversine are indicated in red. Compounds with Oncolines™ profiles having significant correlation, as defined by a Pearson correlation ≥ 0.5, are connected. The analysis shows that reversine clusters with the TTK inhibitors (Figure 8B), which form a unique, separate cluster from the Aurora kinase inhibitors and other mitotic or cell cycle inhibitors [41]. These data indicate that reversine exerts its anti-proliferative activity on cells by inhibiting TTK.

**Figure 9:**
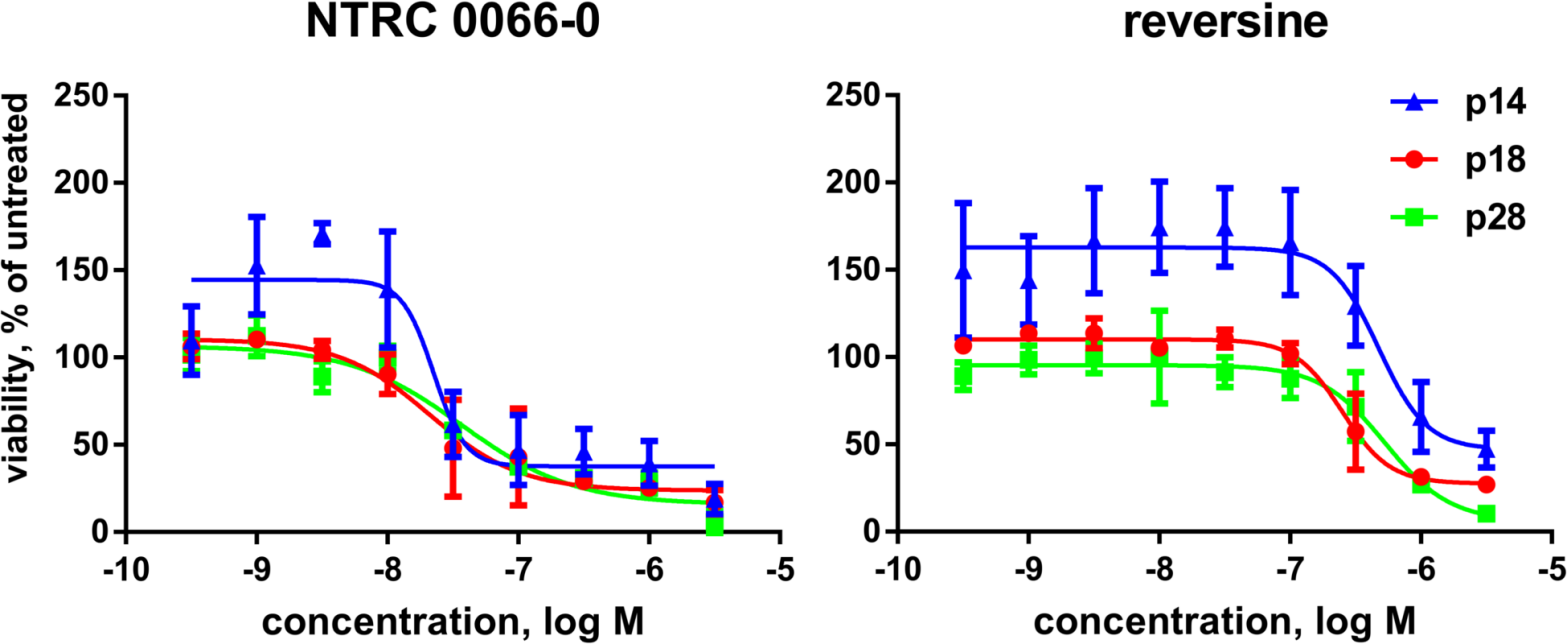
Inhibition of proliferation of patient-derived organoids Dose-response curves of NTRC 0066-0 and reversine in proliferation assays with organoids derived from colorectal carcinoma resections from three different patients: p14 (blue), p18 (red) and p28 (green). Organoids were treated with compound for three days. Curves were fitted using the values of three independent experiments.

Consistent with the anti-proliferative activity on cells (Supplementary Table 1), both NTRC 0066-0 and reversine also inhibited the proliferation of patient-derived organoids derived from colorectal carcinoma resections (Figure 9). NTRC 0066-0 inhibited the proliferation of organoids from three different patients with an average IC_50_ of 27 nM (Figure 9; Supplementary Table 3). This was 10 times more potent than reversine (Supplementary Table 3).

### NTRC 0066-0 only kills proliferating cells

Thus far, we have found that NTRC 0066-0, the most potent and selective TTK inhibitor described in literature, inhibits the proliferation of all human cell lines examined, including two ‘normal’ cell lines, *i.e*., the retinal pigment epithelial cell line hTERT-RPE1 and BJ-5ta skin fibroblasts [18]. These cell lines have been derived from somatic cells by expression of the telomerase catalytic component and lack certain properties of transformed cells [42]. Therefore, these cell lines have often been used as controls to represent ‘normal’, non-transformed cells [42–44]. However, when we performed targeted exome sequence analysis of twenty-five cancer genes, we found that both cell lines contain mutations in at least two known cancer driver genes (Table 2). hTERT-RPE1 contains a missense mutation in the tumor suppressor gene *CDKN2A* and a duplication in the *K-RAS* oncogene (Table 2). BJ-5ta contains two mutations resulting in premature termination of *CDKN2A* and *TP53* (Table 2). The four mutations also have been identified in different patient tumor samples, indicating that they are clinically relevant (Table 2). Since the DNA for sequence analysis was isolated from passage +4 or +5 from the original vials from ATCC, it is likely that these mutations are also present in batches of these cell lines used in other laboratories. Indeed, the two mutations in hTERT-RPE1 were also found in DNA from cell batches of the Max Planck Institute in Martinsried (Germany), who had obtained the cell line from the University of Manchester (U.K.) [45].

**Table 2:** Cancer gene mutations in hTERT-immortalized human primary cell lines

Cell line	Gene	Mutation	Consequence	Frequency^a^
hTERT-RPE1	*CDKN2A*	c.250G > T	missense (p.Asp84Tyr)	5/3484
hTERT-RPE1	*K-RAS*	c.30_35dup	duplication (p.Ala11_Gly12dup)	16/34217^b^
BJ-5ta	*CDKN2A*	c.329G > A	pre-mature termination (p.Trp110*)	10/3484
BJ-5ta	*TP53*	c.976G > T	pre-mature termination (p.Glu326*)	4/18128

^a^Frequency of exact the same point mutations in CDKN2 or TP53 in patient tumor samples or ^b^ insertions at same amino acid position in K-RAS. DNA sequencing data from COSMIC v78.

To study the effect of TTK inhibition on non-dividing cells, that at the same time are relevant for cancer, we determined the effect of NTRC 0066-0 treatment on blood cell samples from ten different pediatric T-cell acute lymphoblastic leukemia (T-ALL) patients (Table 3). The viability of pediatric T-ALL cell samples slowly decreases over a period of three days in tissue culture and treatment with several drugs increases cell death [46]. For example, daunorubicin, an established drug for leukemia, killed all viable cells in the patient samples within a period of three days with IC_50_ < 100 nM (Table 3). Also the proteasome inhibitor bortezomib very effectively kills all cells (IC_50_ < 10 nM). In contrast, treatment of the cell samples with NTRC 0066-0 did reduce viability only at micromolar concentration (IC_50_ ≥ 5 μM) (Table 3). This suggests that TTK inhibition only kills proliferating cells.

**Table 3:** Viability assays with T-ALL patient samples

	Patient samples									
	491	1033	1816	2323	3594	3852	4023	4126	4992	10110
NTRC-0066	5.0E + 3	5.1E + 3	8.1E + 3	7.1E + 3	4.8E + 3	4.9E + 3	5.4E + 3	9.2E + 3	4.9E + 3	4.5E + 3
daunorubicin	80	43	38	80	43	61	57	71	41	42
bortezomib	3	3	2	3	4	4	3	3	3	3
doubling^a^	−1.4	−0.8	−1.3	−2.5	−1.2	−1.0	−5.5	−5.8	−1.8	−0.9

Values are IC_50_ (nM) in three-day assay.

^a^Number of doublings over the timecourse of the assay: log2 of the increase in cell number in the absence of treatment; negative value means net cell dying.

## DISCUSSION

It has been hypothesized that highly CIN tumors are more sensitive to drugs that abrogate the mitotic checkpoint than tumors with stable genomes [2, 16–18]. This hypothesis is based on the assumption that these tumors are dependent on the SAC to cope with the cellular stress caused by CIN. In fact, such tumors often overexpress genes encoding SAC components, such as TTK [27, 47]. However in this study, we report that, contrary to expectation, stable aneuploid cell lines are more sensitive to TTK inhibition than CIN lines, irrespective of the tumor tissue origin of the cell lines. These data suggest that stable aneuploid cells indeed might be more addicted to SAC signaling, potentially because SAC components keep them chromosomally stable. In contrast, pre-existing CIN cells might be intrinsically resistant to TTK inhibitors because they have adapted to cope with chromosomal instability through SAC-independent pathways, such as the efficient shedding of extra chromosomes [7].

There is no single mechanism known that explains how tumor evolution selects CIN cells. However, survival with a constant change of karyotype has been shown to depend on adaptation to metabolic stress [48]. It also has been linked to impairment of the p53 pathway, for instance, in tetraploids of HCT 116 [20, 49, 50]. In addition, CIN fuels a large genetic diversity that can in turn drive the random selection of cells that are able to tolerate high CIN; this ‘lottery’ is not possible in case of aneuploid stable cells. Our results suggest that in order to target cells with a pre-CIN condition, the tolerance threshold must be reduced and reached by *de novo* mis-segregation.

Several different TTK inhibitors have been tested in mouse xenograft models of human cancer cell lines and shown to be efficacious [18–24]. For the first time, we show that TTK inhibitors can also inhibit the proliferation of patient-derived organoids. In contrast to cell lines and organoids, the potent and selective TTK inhibitor NTRC 0066-0 had no effect on the viability of non-dividing cells derived from pediatric T-ALL, which were efficiently killed by chemotherapeutic agents. This lack of activity is consistent with the role of TTK as a SAC kinase, and the fact that TTK is exclusively expressed during mitosis. It should be noted that in toxicity studies in mice, NTRC 0066-0 had no effect on bone marrow or blood cell count, whereas hematological toxicity was seen in the same study upon treatment with docetaxel [18]. Importantly, the clinical application of cell cycle kinases and mitotic kinases, such as CDK4, Aurora or Polo-like kinases, is limited by hematological toxicity [51–53]. In this respect, TTK inhibitors may have an advantage over these other targeted anti-proliferative therapies, although this remains to be shown in the clinic.

A logical next step based on our results would be to look at targeting aneuploid stable tumours and CIN tumours with TTK inhibitors *in vivo*. Previous work aimed to increase chromosomal instability in tumours did not distinguish between CIN levels and stable aneuploidy states [18–22]. On evaluating literature data, we observed that the effect of TTK inhibitors on weight and toxicity is not always provided. TTK inhibition reduced tumour growth in five studies using xenografts models of chromosomally stable or low-CIN cell lines (*i.e*., HCT 116, HeLa, MDA-MB-231 and A2780) [18–24, 54, 55]. Only in three out of five studies the effect of TTK inhibition on mouse body weight is shown [18, 20, 21], which in two cases is present but below 20% weight loss. Ideally, tumour sensitivity should be normalized to the host tolerance for the treatment. Studies to determine the efficacy of NTRC 0066-0 in xenograft models of chromosomal stable tumour cell lines are planned.

We further studied the effect of TTK inhibition in a sub-type of aneuploid cell lines, *i.e*., post-tetraploids. There are conflicting data about the sensitivity of tetraploid and post-tetraploid cells to cytotoxic drugs and targeted inhibitors [34, 36, 37, 56]. One possible explanation is that laboratories use different compounds for the same target. However, we here cleared the controversies surrounding the TTK reference inhibitor reversine, which was previously used to show the selective killing of tetraploid cells by TTK inhibition [36]. Another explanation of conflicting results is that experiments might have occurred in different timeframes. Tetraploid cells have the ability to compensate for deleterious losses or mutations, allowing cells to sample random genomic combinations [33, 57]. In our study, the proliferating post-tetraploid cell lines HPT1, HPT2 and HPT4 did not lose chromosomes after short TTK inhibitor treatment. Instead, treatment with NTRC 0066-0 resulted in increases in chromosome number, cell death and irreversible cell cycle arrest. Since the tetraploids show low level resistance against various cytotoxic drugs and several targeted therapies [34], TTK inhibitors may be a better choice for the eradication of tumor cells that underwent whole genome doubling [34, 36]. Notably, an analysis on The Cancer Genome Atlas (TCGA) Pan-Cancer data set has shown that 37% of cancer underwent whole genome doubling [58].

According to a long-standing hypothesis [31, 32, 59], premalignant stable cells give rise to CIN cancer cells through a tetraploid intermediate. Subsequent loss of chromosomes and tumor suppressor activity causes stable tetraploids to develop into unstable aneuploid tumors [60]. Our results suggest that TTK inhibitor therapy could be a new suitable treatment option in particular at the first two tumor stages, where CIN remains minimal.

## MATERIALS AND METHODS

### Compounds

The TTK inhibitors NTRC 0066-0 [18], MPI-0479605 [20] and Mps-Bay2b [21] were synthesized according to published protocols. Reversine was purchased from Selleck Chemicals; S-trityl-L-cysteine (STLC) from Sigma Aldrich. The source of all other anti-cancer agents is provided in Supplementary Table 2 of Uitdehaag *et al*. [41]. All compounds were stored as powders and freshly dissolved in dimethyl sulfoxide (DMSO) as 10 mM stocks.

### Cell lines

Cancer cell lines and hTERT-RPE1 retinal pigment epithelial cells were purchased from the American Type Culture Collection (ATCC) (Manassas, VA, U.S.A.) from 2011 to 2014 [41] and cultured in ATCC-recommended media. All experiments were carried out within nine passages of the original vials from ATCC who authenticated the cancer cell lines by short tandem repeat analysis. We verified the mutant status of seven frequently mutated cancer genes (*i.e*., *BRAF*, *CDKN2A*, *CTNNB1*, *EGFR*, *KRAS*, *PIK3CA* and *TP53*) by full exome or targeted sequencing. Sequencing results were compared to COSMIC version 75 of the Genomics of Drug Sensitivity in Cancer data base and provided additional authentication of the identity of the cell lines. The isolation and characterization of post-tetraploid clones from HCT 116 has been described previously [34]. The mutation status of the *CDKN2A*, *CTNNB1*, *KRAS* and *PIK3CA* genes were confirmed by sequence analysis in both the HCT 116 parental cell line and post-tetraploid clones. All experiments were carried out within nine passages of the original cell stocks that were transferred from the Max Planck Institute of Biochemistry (Martinsried, Germany) to Netherlands Translational Research Center.

### RNA isolation, cDNA synthesis and real-time PCR

Total RNA was isolated using RNeasy midi kit (Qiagen). cDNA was synthesized using 0.5 μg of RNA using Superscript III reverse transcriptase (Invitrogen) according to manufacturer's instruction. Real-time PCR measurements were performed using SYBR green purchased from Applied Biosystems. Three references genes were used and measurements were done in duplicate. Primers were: β-Actin forward: CAAGAGATGGCCACGGCTGCTTCCA; β-Actin reverse: 5′-GCATGGAGTTGAAGGTAGTTTCG-3′; 18s forward: 5′-AGACAACAAGCTCCGTGAAGA-3′; 18s reverse 5′-CAGAAGTGACGCAGCCCTCTA-3′; HPRT forward: 5′-GACCAGTCAACAGGGGACAT-3′; HPRT reverse: 5′-CCTGACCAAGGAAAGCAAAG-3′. Primers for TTK were previously used [61] and were: TTK forward: 5′-CGCAGCTTTCTGTAGAAATGGA-3′; TTK reverse: 5′-GAGCATCACTTAGCGGAACAC-3′.

### Oligonucleotide transfection

On-TARGETplus SMARTpool: non-targeting siRNAs (D-001810-10-05) and siRNAs targeting Mad2 (L-003271-00-0005) were purchased from Dharmacon. SiRNA transfections were performed 24 hours before the start of experiments using RNAiMAX (Life technologies) according to manufacturer's protocol.

### Immunoblot analysis

Cells were lysed in Laemmli buffer. Samples were boiled and separated by sodium dodecyl sulfate–polyacrylamide gel electrophoresis and transferred to nitrocellulose membranes, blocked with 4% bovine serum albumin (w/v) at room temperature for 1 h, and incubated with primary antibodies at 4°C overnight. The Mad2 (A800-300A, Bethyl) and Actin (I-19, Tebu-bio) antibodies were used at 1/1000 dilution. After incubation with secondary antibody (peroxidase-conjugated goat anti-rabbit, 1:2000 dilution, DAKO) at room temperature for 1 h, the membranes were developed with chemi-luminescence ECL reagent (Amersham, Amersham, UK) and pictures were taken with the ChemiDOC XRS+ (Bio-Rad, Hercules, CA, USA). Analysis was done using ImageJ software (NIH, Bethesda, MD, USA).

### Organoids

The establishment and characterization of the human colon cancer organoids has been described [62]. The organoids were cultured in drops of Geltrex LDEV-Free Reduced Growth Factor Basement Membrane Matrix (Gibco, cat no. A1413202) in DMEM/F12 medium (Thermo), supplemented with 1% (v/v) penicillin/streptomycin (Gibco), 1% Hepes buffer, pH7.5, 1% L-glutamine (Gibco), 1 μg/mL R-spondin (Sigma), 100 ng/mL Noggin (Peprotech), B27 vitamin A-free (Invitrogen, cat no. 12587010), 1 mM n-acetyl cysteine (Sigma), 10 mM nicotinamide (Sigma), 50 ng/mL EGF (BD Biosciences), 500 nM A83-01 (Tocris), 10 μM SB202190 (Sigma), 10 μM Y-27632 (Sigma) and 10 nM Prostaglandin E2 (Cayman). Medium was refreshed every four days and organoids were splitted by treatment with TryPLE Express (Gibco, cat. no. 12604013).

### Patient samples

Written informed consent was obtained from the parents or legal guardians of each T-ALL patient to use excess diagnostic material for research purposes. The study was performed in accordance with the Institutional Review Board of the Erasmus Medical Center Rotterdam (The Netherlands) and in accordance with the Declaration of Helsinki. Leukemic cells were harvested from blood or bone marrow samples and were enriched to ≥ 90% purity.

### Cell proliferation and apoptosis assays

Cell proliferation assays were carried out as described [63] using ATPlite 1step™ (Perkin Elmer, Groningen, The Netherlands). Exposure time was 72 or 120 hours as indicated in the Legends to the Figures. Percentage growth was calculated, relative to the growth of unexposed cells, and relative IC_50_s were fitted using a four-parameter logistics curve (XLfit 5.3, IDBS). For Figure 4A and 7 dose response curves were redrawn in Prism. To compare the inhibitory potency of compounds in proliferation assays with HCT 116 parental and tetraploid clones, pIC_50_ (−^10^logIC_50_) values were compared in three independent experiments. A two-tailed Student's *t*-test was performed to determine whether differences in sensitivity (ΔpIC_50_) were statistically significant. Caspase 3/7 activity of cancer cells was measured using Caspase-Glo 3/7 Assay kit (cat no. G8093, Promega, Madison USA). Viability of T-ALL cell samples was determined using ATPlite and exposure of 72 hours as described previously [46].

### Colony formation assay

HCT 116 parental cells or tetraploids were incubated with 100 nM NTRC 0066-0 or vehicle (DMSO). After 4 days, the cells were washed with phosphate-buffered saline (PBS) and allowed to recover for 24 hours. The remaining cells were collected and plated at a density of 1000 cells per well in a 96-well plate. After ten days, cells were fixed with 96% methanol for 20 minutes, washed with PBS and stained with 0.1% (w/v) crystal violet over-night. Plates were washed in tap water and allowed to dry before scanning and analysis with ImageJ software (NIH, Bethesda, MD, USA).

### Organoids drug sensitivity and viability assay

Organoids were dissociated by TryPLE Express treatment and filtered using a 40 μm nylon cell strainer (Falcon). The organoids were re-suspended in Geltrex at a density of 250,000 cells/mL and seeded in the wells of 96-well plates (Corning, cat no. 3903) at 10 μl per well. Compounds were added four days after plating and 72 hours later viability was determined using Cell-Titer Glo 3D Cell Viability Assay (Promega, Madison USA). Experiments were performed in three biological replicates and technical triplicates were averaged per experiment.

### Time-lapse microscopy

HCT 116 diploid and tetraploids stably expressed green fluorescent protein-tagged histone 2B (H2B-GFP). All other cell lines were pre-incubated with SiR-DNA (Spirochrome, Switzerland) in order to visualize DNA. Cells were cultured in Leibovitz L15 CO_2_-independent cell culture medium in 6-well glass bottom chamber (LabTek Corp., Australia) and synchronized by treatment with 2 mM thymidine. After 24 hours of synchronization, and 4 hours before the start of the imaging experiment, medium was replaced by medium without thymidine. Cells treated with NTRC 0066-0 or vehicle (DMSO) were imaged every 5 min in a heated chamber at 37°C, using a ×40 NA 0.95 air objective on an IX71 microscope (Olympus) controlled by SoftWoRx 6.0 software (Applied Precision). Image Z-stacks were acquired with 3-μm intervals using a sCMOS camera (DeltaVision RT; Applied Precision, GE Healthcare, Issaquah, WA) and processed using ImageJ software (NIH, Bethesda, MD, USA). Chromosome mis-segregation phenotypes co-occurred and many combinations existed. For clarity, all the events were scored and included: more than two phenotypes, mitotic slippage, DNA bridge, micro-nuclei and multipolar spindle.

### Karyotype analysis

Cells were plated in a 10 cm^2^ dish and allowed to adhere overnight. To synchronize cells, 2 mM of thymidine was added, and after 24 hours the medium was changed to medium without thymidine and NTRC 0066-0 or vehicle. The next day, the cells were washed with PBS and treated overnight with 100 ng/mL nocodazole (Supplementary Figure 4). Cells blocked in mitosis were collected by mitotic shake off and incubated for 30 min at 37°C with 10 mL of hypotonic solution (1:4, media: tap water) and centrifuged for 8 min at 1000 rpm. The mitotic cells were fixed overnight in fresh Carnoy solution (1:4, acetic acid: methanol). The next day, cells were suspended in a small volume of fresh Canoy solution with 300 nM 4′,6-diamidino-2-phenylindole (DAPI). With distance, 3 drops of the cells/fixative were realized onto a microscope glass slide. Slides were allowed to dry and mounted with Prolong antifade (Thermo, cat no. P36934). Chromosome spreads were imaged using the Metafer4/MSearch automated metaphase finder system (MetaSystems, Germany), equipped with an AxioImager Z2 microscope (Carl Zeiss, Germany). After scanning metaphase preparations at 10x magnification, high-resolution images of metaphases were acquired using a ‘Plan-Apochromat′ 63×/1, 40 oil objective. For the tetraploid cell lines the imaging was done using delta vison (DeltaVision RT; Applied Precision, GE Healthcare, Issaquah, WA). For both, the analysis was done using ImageJ software (NIH, Bethesda, MD, USA).

### Cell cycle analysis

Cells were plated in a 10 cm^2^ dish and allowed to adhere overnight. To synchronize cells, 2 mM of thymidine was added, and after 24 hours the celsl were collected (Supplementary Figure 4) and fixed in ice cold 70% ethanol for at least 2 hours at 4°C. Cells were incubated in Propidium Iodide (PI) staining solution for 10 min at 37°C. PI staining solution: 0.1% (v/v) Triton X-100, 10 μg/mL PI (Molecular Probes), and 100 μg/mL DNase-free RNase A (Sigma) in PBS. Samples were analyzed on Becton Dickinson FACSCalibur analyser and using Flowing software 2.

### Surface plasmon resonance

Binding kinetics of NTRC 0066-0 and reversine was determined by surface plasmon resonance using Biacore T200 (GE Healthcare) using bacterially expressed TTK kinase domain and Sf9 insect cell-expressed Aurora B (Carna Biosciences, Inc., Kobe, Japan) as described [18, 41].

## SUPPLEMENTARY MATERIALS FIGURES AND TABLES



## Abbreviations

CIN: chromosome instability
DMSO: dimethyl sulfoxide
SAC: spindle assembly checkpoint
PBS: phosphate-buffered saline
T-ALL: T-cell acute lymphoblastic leukemia.
