# Supplementary material for: Stable aneuploid tumors cells are more sensitive to TTK inhibition than chromosomally unstable cell lines
Source: Oncotarget. 2017 Mar 15;8(24):38309–25. doi: 10.18632/oncotarget.16213 (PMC5503534; doi:10.18632/oncotarget.16213)
Supplement: Supplementary file 1 [file oncotarget-08-38309-s001.pdf]

Stable aneuploid tumors cells are more sensitive to TTK inhibition than chromosomally unstable cell lines

Supplementary Materials

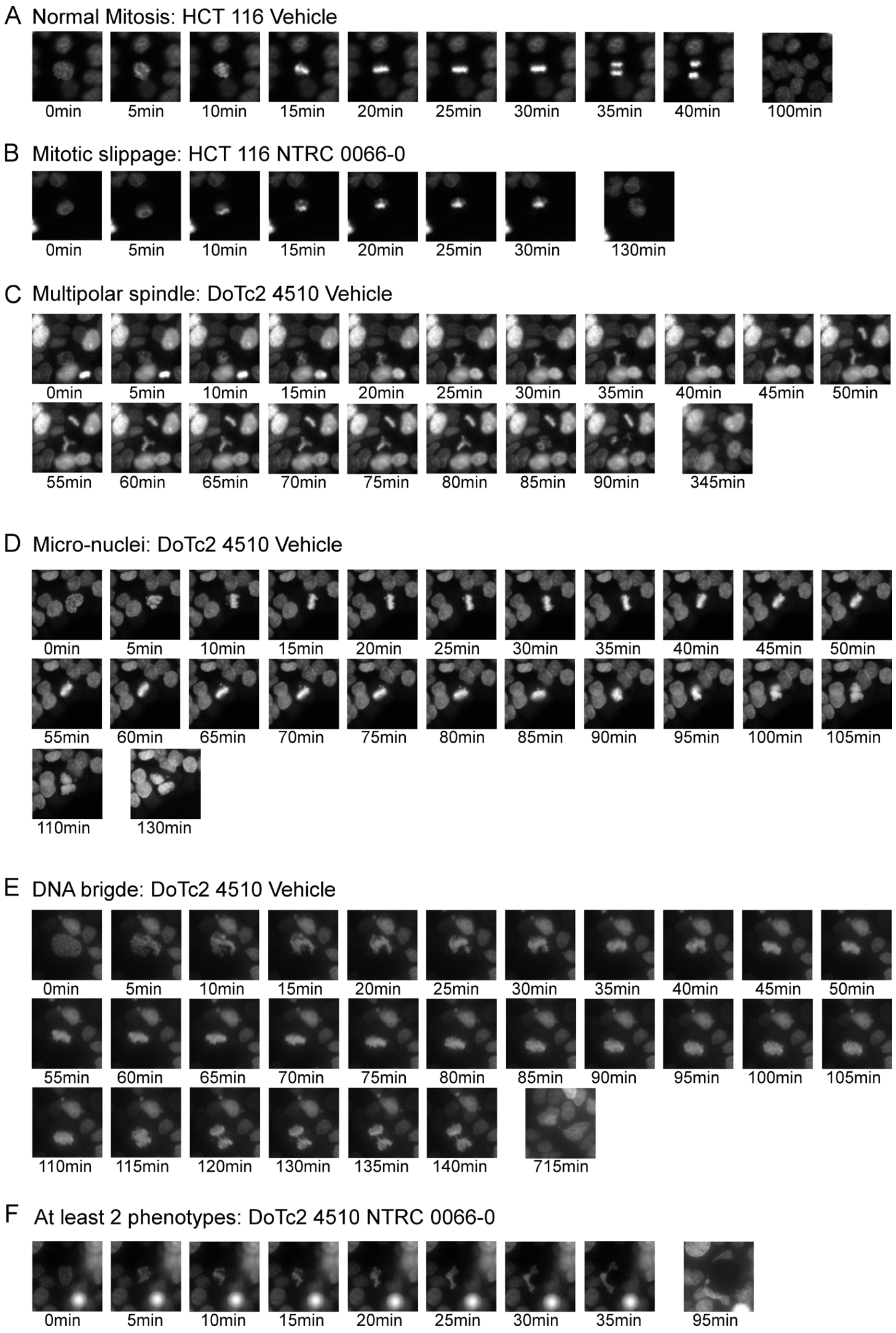

Supplementary Figure 1: Mitotic timing and mis-segregation phenotypes. Representative images from time-lapse microscopy. Examples show the length of mitosis and the observed mis-segregation phenotypes. (**A**) Normal mitosis: HCT116 Vehicle (**B**) Mitotic slippage: HCT 116 NTRC 0066-0 (**C**) Multipolar Spindle: DoTc2 4510 Vehicle (**D**) Micro- nuclei: DoTc2 4510 Vehicle (**E**) DNA bridge: DoTc2 4510 Vehicle (**F**) At least 2 phenotypes, mitotic slippage and DNA bridge: DoTc2 4510 NTRC 0066-0.

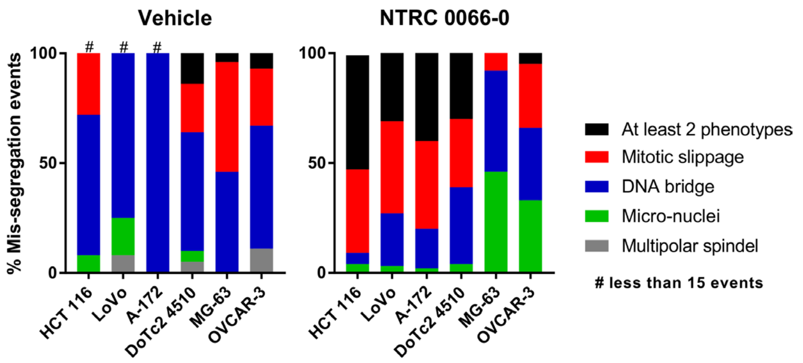

Supplementary Figure 2: Mis-segregation phenotypes breakdown. Resistant or sensitive cells lines treated with Vehicle or NTRC 0066-0.

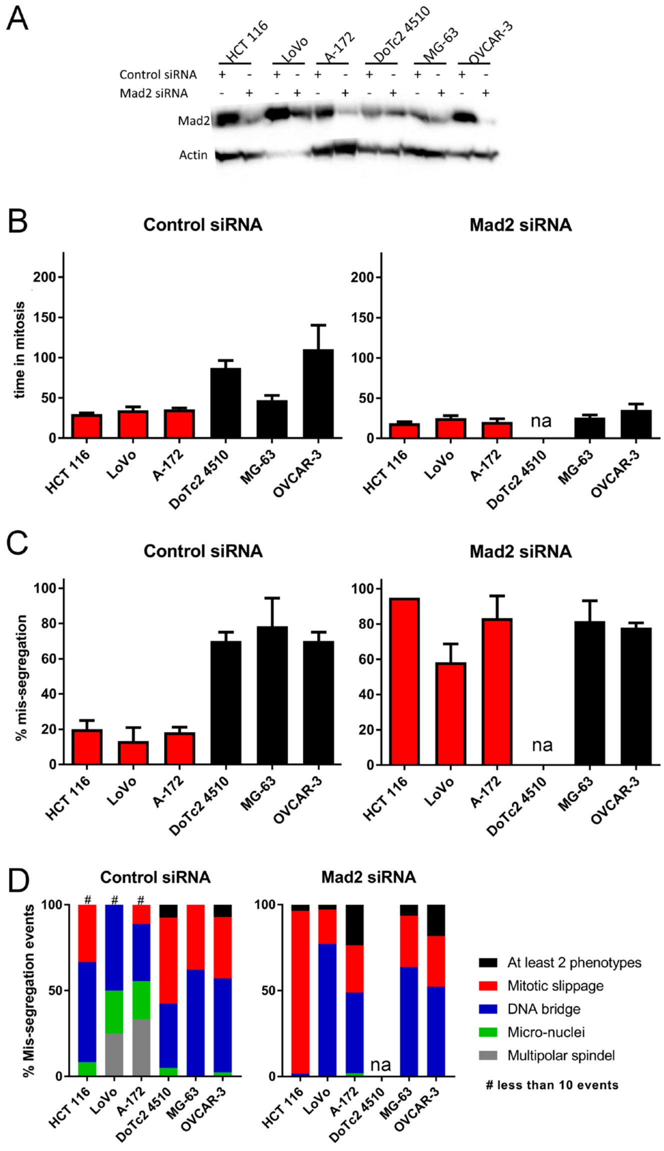

Supplementary Figure 3: Mitotic timing and mis-segregation upon reduction of Mad2 level. (**A**) Representative immuno-blot showing the expression of the Mad2 protein in six cell lines treated with control siRNA or Mad2 siRNA. Actin Immuno-blot serves as loading control. Mad2 expression was reduced in all cell lines expect DOTC 4510. The experiment has been performed three times. (**B**–**D**) Time lapse microscopy analysis of NTRC0066-0 sensitive (red) and resistant (black) cell lines treated with control siRNA (left) or siRNA for Mad2 (right). The bar graphs represent means and standard deviation calculated from three independent experiments. For one experiment 25 cells were quantified on average. na = non-applicable. A) Time in mitosis (from nuclear envelope breakdown to anaphase onset). B) Percentage of cells dividing with mis-segregation. D) Mis-segregation phenotypes breakdown.

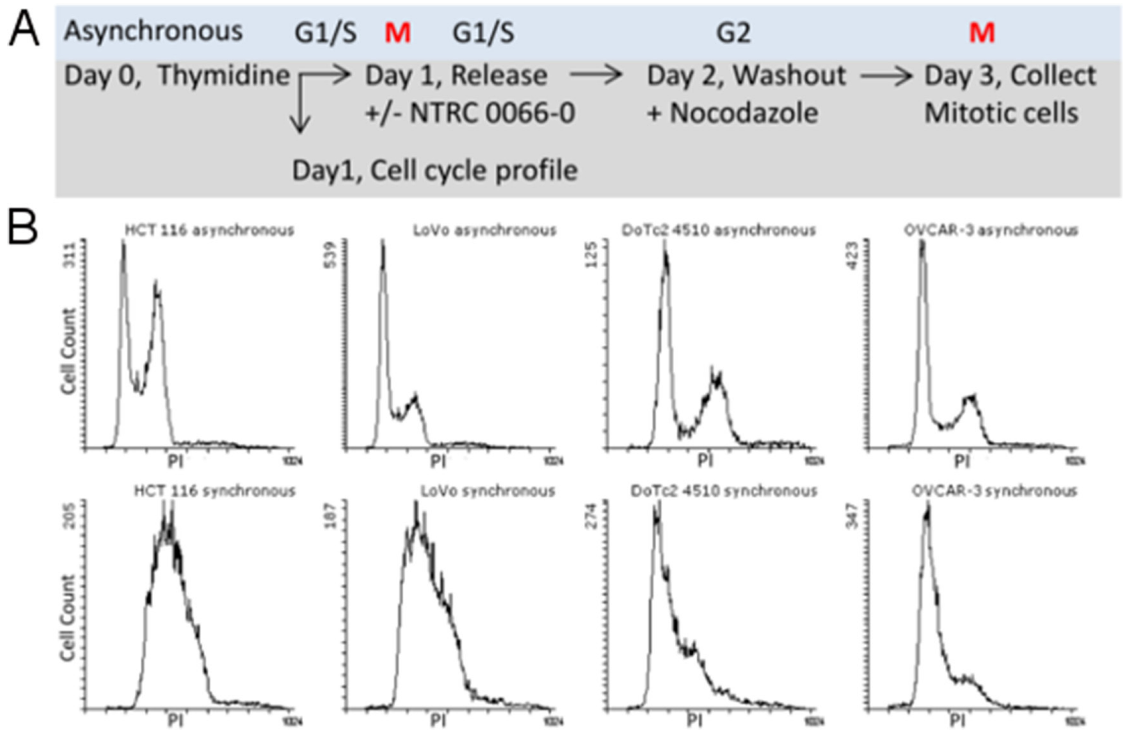

Supplementary Figure 4: Experimental set-up for karyotype analysis. (**A**) Scheme representing the experimental steps. Asynchronous cells were treated with Thymidine for 24 hours. The cells arrest in G1/S. Simultaneously, thymidine was washed-out and NTRC 0066-0 (100 nM) or vehicle were added. It allows cells to resume the cell cycle and go thought mitosis (M). The progeny can start a new cell cycle (G1/S). The next day, before the start of the second mitosis, in G2, NTRC 0066-0 or the vehicle were washed-out and Nocodazole was added. Nocodazole trapped the cells at the second mitosis and cells were collected for metaphase spreads. (**B**) Cell cycle histogram showing on the X axis Propidium Iodide (PI) staining intensity and on the Y axis the cell counts. On the upper panel, untreated cells and on the lower panel are cells treated with Thymidine for 24 hours. The upper panel shows asynchronous HCT 166, LoVo, DoTC2 4510 and, OVCAR-3 cell lines, with a clear mitotic population. The lower panel shows synchronous population of the four cell lines.

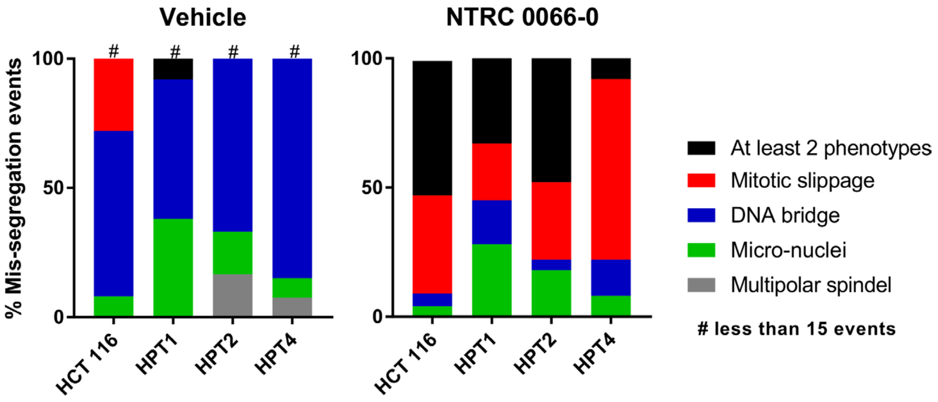

Supplementary Figure 5: Mis-segregation phenotypes breakdown. HCT 116, HPT1, HPT2 and, HPT4 cells lines treated with Vehicle or NTRC 0066-0.

Supplementary Table 1: Anti-proliferative activity (IC_{50}, in nM) of TTK inhibitors in 3 and 5 day cancer cell line proliferation assays

	NTRC 0066-0		MPI-047605	Bay 2b	reversine
**Cell line**	3 days	5 days	3 days	5 days	5 days
HCT 116	37	41	234	1387	175
LoVo	40	35	220	1434	113
A-172	51	34	198	1628	158
DoTc2 4510	117	225	8392	2559	416
MG-63	135	260	899	3757	434
OVCAR-3	897	268	7328	5486	441
[a]average IC_{50}	[b] 67	98	[b] 448	1820	239

[a]geometric average of IC_{50} in 66 cancer cell lines, except [b] in 44 cell lines [1, 2]

1. Maia AR, de Man J, Boon U, Janssen A, Song JY, Omerzu M, Sterrenburg JG, Prinsen MB, Willemsen-Seegers N, de Roos JA, van Doornmalen AM, Uitdehaag JC, Kops GJ, et al. Inhibition of the spindle assembly checkpoint kinase TTK enhances the efficacy of docetaxel in a triple-negative breast cancer model. Ann Oncol. 2015; 26:2180–92. doi: 10.1093/annonc/mdv293.

2. Uitdehaag JC, de Roos JA, Prinsen MB, Willemsen-Seegers N, de Vetter JR, Dylus J, van Doornmalen AM, Kooijman J, Sawa M, van Gerwen SJ, de Man J, Buijsman RC, Zaman GJ. Cell panel profiling reveals conserved therapeutic clusters and differentiates the mechanism of action of different PI3K/mTOR, Aurora kinase and EZH2 inhibitors. Mol Cancer Ther. 2016. doi: 10.1158/1535-7163.MCT-16-0403.

Supplementary Table 2: Drug sensitivity analysis in HCT116 diploid and tetraploids

A: Differences based on potency (IC_{50})

		HCT 116	HPT1	HPT2	HPT4	HCT 116 vs HPT1, -2 and -4		
**Compound**	**Target**	**IC_{50} (nM)**	**IC_{50} (nM)**			**ΔpIC_{50}**	**Phenotype**	***p*-value**
doxorubicin	topoisomerase II	31	69	130	83	0.46	resistant	0.0002
NTRC 0066-0	TTK	44	40	44	44	−0.01	sensitive	0.844
reversine	TTK	242	208	253	159	−0.08	sensitive	0.463
Mps-Bay2b	TTK	1581	1894	1798	1578	0.04	resistant	0.554
MPI-479605	TTK	233	208	199	191	−0.07	sensitive	0.580
MLN-8054	Aurora A kinase	715	1330	1553	1196	0.28	resistant	0.001
GSK-1070916	Aurora B/C kinase	14	16	1700	1679	1.41	resistant	0.041
volasertib	PLK1	25	26	45	49	0.19	resistant	0.062
STLC	kinesin Eg5	711	2042	1340	1401	0.34	resistant	0.0002

IC_{50} are geometric averages of three experiments. ΔpIC50 are the average log shift in sensitivity between parental and tetraploids. 'resistant' indicates that tetraploids are resistant. Student's *t*-test *p*-values are based on a two tailed distribution of three values. Bold indicates when differences were statistically significant, $p < 0.05$.

B: Differences in sensitivity based on efficacy (% effect)

		HCT 116	HPT1	HPT2	HPT4	HCT 116 vs HPT1, -2 and -4		
**Compound**	**Target**	**Eff. (%)**	**Efficacy (%)**			**Δeff. (%)**	**Phenotype**	***p*-value**
doxorubicin	topoisomerase II	97	99	100	100	3	sensitive	0.013
NTRC 0066-0	TTK	80	59	36	48	−33	resistant	0.001
reversine	TTK	77	58	44	33	−32	resistant	0.001
Mps-Bay2b	TTK	87	66	49	52	−31	resistant	0.0002
MPI-479605	TTK	72	52	46	51	−22	resistant	0.0002
MLN-8054	Aurora A kinase	85	61	52	56	−28	resistant	0.00001
GSK-1070916	Aurora B/C kinase	61	32	100	100	16	sensitive	0.456
volasertib	PLK1	98	82	65	84	−21	resistant	0.004
STLC	kinesin Eg5	99	90	93	95	−6	resistant	0.003

% effect values (Eff.) are averages of three experiments.

Supplementary Table 3: Sensitivity of organoids to NTRC 0066-0 and reversine in proliferation assays

	NTRC 0066-0		reversine		difference	
**Organoid**	**IC_{50} (nM)**	**pIC_{50}**	**IC_{50} (nM)**	**pIC_{50}**	**ΔpIC50**	***p*-value**
p14	22.5	7.65	493.0	6.33	1.34	**0.0004**
p18	22.1	7.68	284.9	6.57	0.96	0.0504
p28	37.0	7.43	757.1	6.19	1.22	**0.0029**
All		7.59		6.36	1.17	**0.0008**

Anti-proliferative activity is expressed as IC_{50} and pIC_{50}. Results are shown from three different independent experiments and geometric average of pIC_{50}. Difference in sensitivity is expressed as ΔpIC_{50}. Bold p-values indicate statistically significant ($p < 0.05$).
